# Mapping of the cumulative β-ray dose on the ground surface surrounding the Fukushima area

**DOI:** 10.1093/jrr/rrv056

**Published:** 2015-10-31

**Authors:** Satoru Endo, Tsuyoshi Kajimoto, Kenichi Tanaka, Thanh T. Nguyen, Gohei Hayashi, Tetsuji Imanaka

**Affiliations:** 1 Quantum Energy Applications, Graduate School of Engineering, Hiroshima University, 1-4-1 Kagamiyama, Higashi–Hiroshima 739-8527, Japan; 2 Institute of Development, Aging and Cancer, Tohoku University, Seiryo-machi 4-1, Aoba-ku Sendai, 980-8575, Japan; 3 Kyoto University Research Reactor Institute 2, Asashiro-Nishi, Kumatori-cho, Sennan-gun, Osaka 590-0494, Japan

**Keywords:** Fukushima Daiichi Nuclear Power Plant accident, β-ray dose, radiotellurium, radioiodine, radiocesium

## Abstract

A large amount of the fission products released by the Fukushima Daiichi Nuclear Power Plant (FDNPP) accident on 11 March 2011 was deposited in a wide area from Tohoku to northern Kanto. A map of the estimated cumulative β-ray dose (70 μm dose equivalent) on the soil surface for one year after the FDNPP accident has been prepared using previously reported calculation methods and the 2-km mesh survey data by MEXT. From this map of estimated dose, areas with a high cumulative β-ray dose on the soil surface for one year after the FDNPP accident were found to be located in the Akogi-Teshichiro to Akogi-Kunugidaira region in Namie Town, and in the southern Futaba Town to the northern Tomioka Town region. The highest estimated cumulative β-ray dose was 710 mSv for one year at Akogi-Teshichiro, Namie Town.

## INTRODUCTION

The nuclear accident at the Fukushima Daiichi Nuclear Power Plant (FDNPP) occurred after the enormous earthquake and associated tsunami on 11 March 2011. A large amount of fission products was released and deposited over a wide area from the Tohoku region to the northern Kanto region [1–4]. The deposited radionuclides were mainly ^129m^Te, ^129^Te, ^131^I, ^132^Te, ^132^I, ^134^Cs, ^136^Cs and ^137^Cs. These radionuclides emit both γ rays and β rays. Because β rays do not contribute to the effective dose, dose evaluations have been focused on γ rays. However, β rays contribute to the skin dose for humans, the whole-body dose for small insects, and the total dose for plant leaves.

In our previous publication, the time variation in the β-ray dose rate and the cumulative β-ray dose as 70 μm dose equivalent were estimated for the conditions of an initial ^137^Cs deposition of 1000 kBq/m^2^, using a Monte Carlo calculation [5]. In the current study, the deposition ratios of ^129m^Te, ^129^Te, ^131^I, ^132^Te, ^132^I and ^134^Cs to ^137^Cs were taken into account, values for which ratios were mainly taken from the Iitate Village contamination study [5]. For example, the ^131^I/^137^Cs ratio was assumed to be 9.2 at the time of deposition [4]. However, the ^131^I/^137^Cs ratio has a range of values for the various areas between the northwestern region and the southern region of the FDNPP [1, 2].

In addition, the Ministry of Education, Culture, Sports, Science and Technology (MEXT) conducted a 2-km mesh contamination study from June to August 2011 [6]. This study started three months after the main deposition occurred on 15 March 2011. Therefore, the short-half-life radionuclides, such as ^132^I and ^132^Te (half-life: 3.204 days), had already decayed out. ^131^I was also decayed by a factor of 2000 due to its short half-life (8.021 days). In the MEXT study, ^131^I radioactivity was detected in only 19% of 2181 soil sampling locations. Consequently, the ^131^I/^137^Cs ratio is available for only 415 locations in the Fukushima prefecture.

The purpose of this paper was to evaluate the cumulative β-ray dose (70 μm dose equivalent) for one year after the FDNPP accident on the ground surface and to create a β-ray dose map of contaminated areas in the Fukushima prefecture, using our previous β-ray calculation method [5] coupled with the MEXT 2-km mesh soil data [6].

## MATERIALS AND METHODS

### Calculation technique for the cumulative soil surface β-ray dose for one year

A previously published β-ray dose calculation technique [5, 7, 8] was used in this study. The transport of β-rays was simulated with Monte Carlo N-Particle transport code version 4C (MCNP-4C) [9]. Beta-ray sources were uniformly distributed in a surface soil layer of 5-mm thickness. Beta-ray energy spectra were used for the radionuclides: ^129m^Te, ^129^Te, ^131^I, ^132^Te, ^132^I, ^134^Cs and ^137^Cs [5]. Energy deposition in the air cell is accumulated as a function of height from the soil surface. The initial β-ray dose rate for each of seven radionuclides was calculated. Then, seven values of β-ray dose rates for the radionuclide *i* at the time of deposition (${\dot{D}}_{i}^{0}$) were combined according to the deposition ratio with that of ^137^Cs: *f_i_*, where *f_i_* is the radionuclide ratio: ^129m^Te/^137^Cs, ^129^Te/^137^Cs, ^131^I/^137^Cs, ^132^Te/^137^Cs, ^132^I/^137^Cs, ^134^Cs/^137^Cs and ^137^Cs/^137^Cs. In order to estimate the time variation, each β-ray dose rate component was reduced by the time after deposition according to each half-life time. The cumulative β-ray dose for one year was calculated by integration of the time after deposition. In the previous publication, the *f_i_* was assumed to be 1: 0.7: 9.2: 8.3: 8.3: 1: 1, where *i* means ^129m^Te, ^129^Te, ^131^I, ^132^Te, ^132^I, ^134^Cs and ^137^Cs [5]. In our study, in the calculation process for the cumulative β-ray dose, radionuclide ratios of ^131^I/^137^Cs and ^129m^I/^137^Cs were treated as two parameters of: *r_I_* and *r_T_*, respectively. Also, ^129^Te/^137^Cs and ^132^I/^137^Cs ratios were scaled to the ^129m^Te/^137^Cs ratio by factors of 0.7 and 8.3, respectively. Therefore, a relation of ^129^Te/^137^Cs = 0.7 x ^129m^Te/^137^Cs and ^132^I/^137^Cs = 8.3 x ^129m^Te/^137^Cs were used here, respectively. Consequently, the deposition ratio of *f_i_* was set to *r_T_*: (0.7·*r_T_*): *r_I_* : (8.3·*r_T_*): (8.3·*r_T_*): 1: 1. The β-ray dose rate (${\dot{D}}_{i}^{0}$) and the cumulative β-ray dose on the ground surface for one year (*D_A_*) are under conditions of an initial deposition density of ^137^Cs. The cumulative dose of *D*(*r_I_ , r_T_*) for one year at unit deposition of ^137^Cs (kBq/m^2^) can be written as follows:

Eq. 1 $$\dot{D}(r_{I},r_{T},t)=\sum_{i}\;f_{i}\cdot {\dot{D}}_{i}^{0}{\left(\frac{1}{2}\right)}^{\frac{t}{T_{i}}},$$

Eq. 2 $$D(r_{I},r_{T})=\int_{0}^{1year}\dot{D}(r_{I},r_{T},t)dt,$$

where *T_i_* is the half-life of radionuclide *i*, and *r_I_* and *r_T_* are ^131^I/^137^Cs and ^131^I/^137^Cs ratios, respectively. After this calculation, the relationships between the cumulative soil surface β-ray dose for one year and conditions of deposition density of ^137^Cs were determined. The cumulative soil surface β-ray dose for one year (*D_A_*) was calculated by:

Eq. 3 $$D_{A}=D(r_{I},r_{T})\cdot A_{{\;}^{137}Cs},$$

where $A_{{\;}^{137}Cs}$ is the ^137^Cs deposition density (kBq/m^2^) taken from the MEXT 2-km mesh soil contamination data [6]. The dose conversion factor from Gy to Sv for β-rays was assumed to be 1 in this analysis.

### ^129m^Te/^137^Cs ratio interpolation

**Fig. 1.**
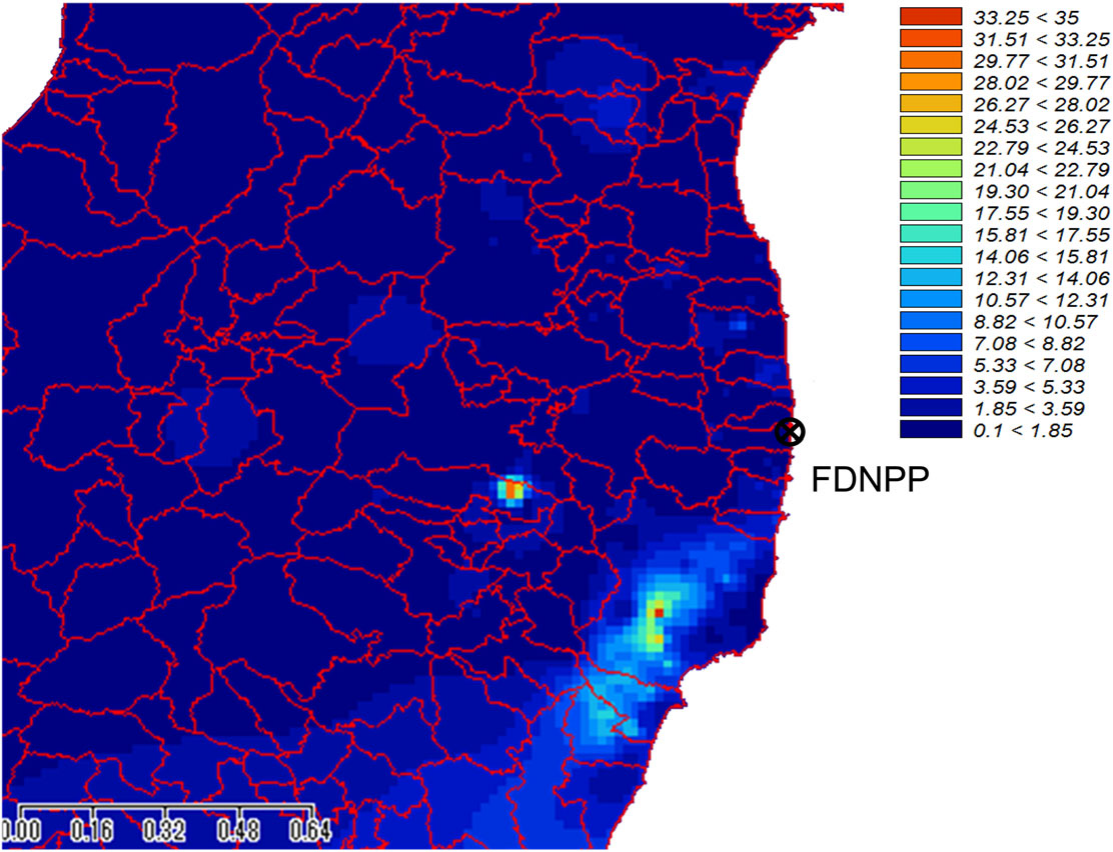
Map of ^129m^Te/137Cs ratio of the MEXT data [6] interpolated with a multilevel B spline interpolation by SAGA-GIS [10].

The ^129m^Te/^137^Cs ratio was obtained from the MEXT data (which includes 2181 sampling locations) at 797 locations. However, both ratios of ^129m^Te/^137^Cs and ^131^I/^137^Cs were obtained at only 175 locations. At the locations without ^129m^Te/^137^Cs data, ^129m^Te/^137^Cs data were interpolated with geographic information techniques (GIS): a multilevel B spline interpolation by SAGA-GIS [10]. The resultant ^129m^Te/^137^Cs map is shown in Fig. 1.

## RESULTS AND DISCUSSION

**Fig. 2.**
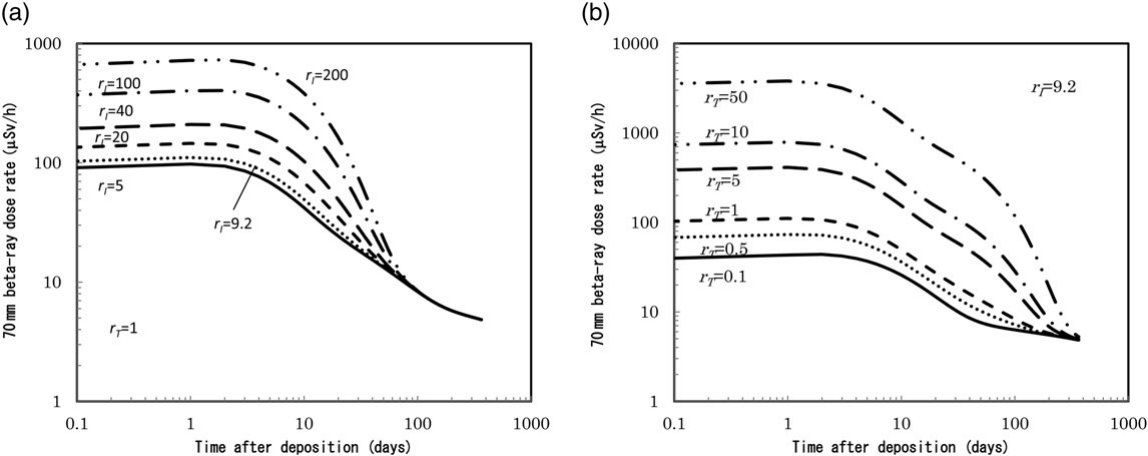
Beta-ray dose rate, μSv h^-1^, on soil surface as a function of time after deposition for variable ^131^I/^137^Cs and ^129m^Te/^137^Cs. (**a**) ^129m^Te/^137^Cs = 1 with ^131^I/^137^Cs = 5, 9.2, 20, 40, 100 and 200. (**b**) ^131^I/^137^Cs = 9.2 with ^129m^Te/^137^Cs = 0.1, 0.5, 1, 5, 10 and 50.

The time dependence of the β-ray dose (70-μm dose equivalent) rate on the ground surface is shown in Fig. 2a for a fixed value of *r_T_* = 1.0, with parameter values of *r_I_* = 5, 9.2, 20, 40, 100 and 200. The ^131^I contribution diminishes about 80 days after deposition due to the decay from Fig. 2a. Figure 2b shows the time dependence of the β-ray dose rate for a fixed *r_I_* of 9.2 with various *r_T_* of 0.1, 0.5, 1, 5, 10 and 50. In case of *r_T_* being >5, small increases in the β-ray dose appear from 20 days. This increase is caused by the contribution of β-rays from ^129,129m^Te nuclides, which have a half-life of 33.6 days. For detailed calculation methods, please refer to the previous publication [5].

**Fig. 3.**
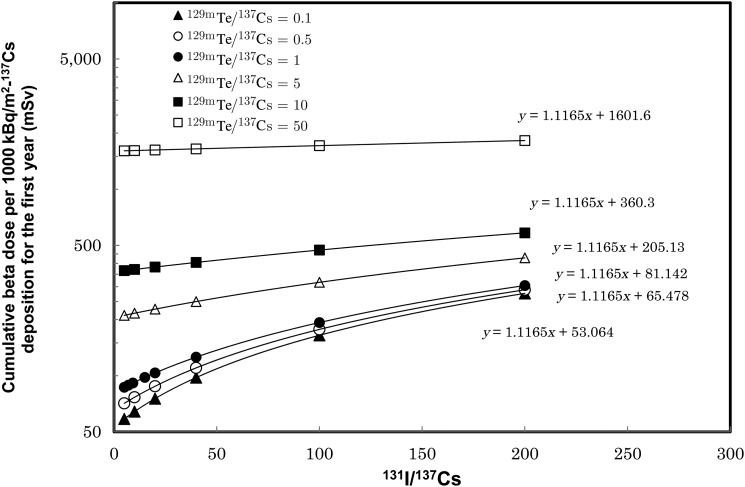
Cumulative β-ray dose per ^137^Cs deposition of 1000 kBq/m^2^ for the first year as a function of ^131^I/^137^Cs and ^129m^Te/^137^Cs ratio.

**Fig. 4.**
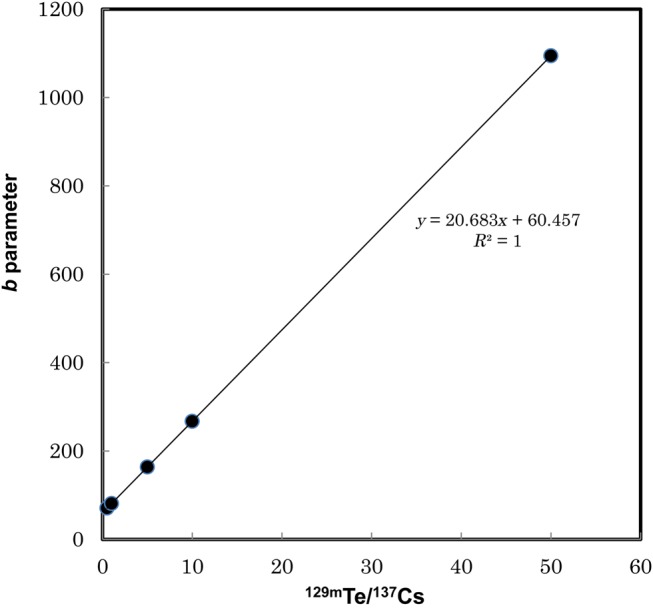
Fitted parameter *b* as a function of ^132^I/^137^Cs ratio.

The cumulative β-ray dose on the ground surface can be obtained by integrating the time-dependent dose rate as Eq. 2. Results of cumulative β-ray dose calculation for various sets of *r_I_* and *r_T_* values are plotted in Fig. 3, respectively. The cumulative dose per ^137^Cs deposition of 1000 kBq/m^2^ is increasing with the ^131^I/^137^Cs ratio. The least square fitted function was determined to be *D*(*r_I_, r_T_*) = 1.1165 *r_I_* + *b*, as shown in Fig. 3. The fitted parameter values of *b* for several values of *r_T_* are listed in Table 1. The fitted parameter: *b* was re-fitted by linear function and determined to be *b*(*r_T_*) = 31.032 *r_T_* + 50.009*.* The fitted result is shown in Fig. 4. Finally, the cumulative β-ray dose on the ground surface per initial^137^Cs deposition for one year, *D*(*r_I_, r_T_*), can be expressed as a function of *r_I_* and *r_T_* as:

Eq. 4 $$D(r_{I},\;r_{T})=1.1165\;\cdot r_{I}+31.032r_{T}+50.009.$$

**Table 1.** Fitted parameter *b* for ^132^I/^137^Cs of 0.1, 0.5, 1, 5, 10 and 50

^132^I/^137^Cs	Parameter *b*
0.1	53.064
0.5	65.478
1	81.142
5	205.13
10	360.3
50	1601.6

The cumulative soil surface β-ray dose for one year was calculated for 415 MEXT sampling locations using Eq. 4. The representative 72 locations selected from 415 locations are listed in Table 2. The calculated results show that higher cumulative β-ray doses appear around the Akogi region in Namie Town and from Futaba Town to northern Tomioka Town. The values for cumulative soil surface β-ray dose were estimated to be 710 mSv at Namie-Akogi-Teshichiro, 477 mSv at Namie-Akogi-Kunugidaira, 246 mSv at Futaba-Ishiguma and 620 mSv at Tomioka-Osuge. Also, the southern Iitate Village had a relatively high cumulative β-ray dose of 100–150 mSv. In Fukushima City, the cumulative soil surface β-ray dose around the eastern region was estimated to be 20–60 mSv higher than that around the western region (4–10 mSv). On the other hand, areas with a high ^131^I/^137^Cs ratio of 69 ± 39 (maximum: 285) around Iwaki City showed a relatively low deposition density of ^137^Cs of 20–50 kBq/m^2^; thus, the cumulative β-ray dose showed slightly lower values: ∼1–24 mSv.

**Table 2.** Cumulative soil surface β-ray dose for one year at representative 72 locations selected from the calculated results of 415 locations using the 2-km mesh soil deposition density (kBq/m^2^) by MEXT [6]

Location	Longitude	Latitude	^131^I	^137^Cs	^129m^Te	^131^I/^137^Cs	^129m^Te/^137^Cs	*D(r_I_*,*r_T_)* (mSv)	*D_A_* (mSv)
Iwaki City, Hisanohama	37.17292	140.9993	0.6	59		26.5	3.8	199	11.7
Iwaki City, Yamada	36.92822	140.7411	0.86	44	88	50.9	11.5	465	20.4
Iwaki City, Yotsukura	37.10747	140.9664	0.76	35	8.8	56.5	2.3	187	6.6
Iwaki City, Taira	37.03022	140.9233	0.82	26	6.9	82.1	1.7	199	5.2
Otama Village	37.54375	140.332	0.41	130	16	8.2	1.1	92.5	12.0
Kuwaori Town, Yachi	37.86128	140.5363	0.32	83	18	10.0	1.3	103	8.5
Kuwaori Town, Kamikori	37.84794	140.5284	1	200	36	13.0	1.3	105	20.9
Kunimi Town, Okido	37.89039	140.5723	0.69	110	18	16.3	1.1	103	11.3
Kawamata Town, Yamakiya	37.583	140.7186	3.6	870	190	10.8	1.3	104	90.0
Kawamata Town, Akiyama	37.69419	140.5603	0.25	51	11	12.8	1.3	105	5.4
Kawamata Town, Kotsunagai	37.63153	140.6581	0.46	81	22	14.8	1.4	111	9.0
Date City, Ryozan	37.784	140.6704	0.15	49		8.0	1.5	105	5.1
Date City, Yanagawa	37.85194	140.5667	0.84	180	35	12.1	1.2	101	18.2
Date City, Miyoda	37.74461	140.6127	1.7	320	52	13.8	1.2	104	33.2
Aizuwakamatsu City, Oto	37.37189	139.9251	0.49	4.5		283	1.3	421	1.9
Kagamiishi Town	37.25781	140.3391	0.25	76		8.6	1.6	110	8.3
Tenei Village	37.22039	140.2581	0.35	140	21	6.5	1.0	89.8	12.6
Koriyama City, Hiwada	37.45531	140.3889	0.12	170	5.3	1.8	0.7	75.3	12.8
Koriyama City, Narukami	37.39411	140.3381	0.49	230		5.5	1.0	87.2	20.1
Sukagawa City, Moriya	37.33333	140.2456	0.089	200		1.2	1.4	94.2	18.8
Sukagawa City, HokotsukiFurudate	37.26972	140.2694	0.6	130	20	12.0	1.0	95.3	12.4
Saigo Village	37.17011	140.2939	0.45	44	7.7	26.6	1.2	118	5.2
Kodono Town	37.07803	140.5705	0.04	33	0.82	3.2	0.4	66.5	2.2
Hirata Village	37.23067	140.5666	0.35	8.1		112	1.5	228	1.8
Katsurao Village, Ochiai-Karogawa	37.48575	140.8077	0.68	160		11.1	1.2	100	16.0
Katsurao Village, Katsurao	37.53778	140.7802	7.3	1600		11.9	1.2	102	163
Hirono Town, Oriki	37.19908	141.0019	2.1	250		21.9	4.5	215	53.7
Hirono Town, Yusuji	37.18967	140.9977	1.3	55	42	61.5	4.4	257	14.1
Kawauchi Village, Shimokawauchi	37.27697	140.8097	0.7	480	64	3.8	0.9	81.2	39.0
Kawauchi Village, Kamikawauchi	37.30375	140.7622	0.15	52	1.8	7.5	0.3	69.5	3.6
Futaba Town, Ishikuma	37.43553	140.954	31	1700	320	47.4	1.3	145	246
Okuma Town, Kumakawa	37.39225	141.0124	32	1700	250	49.0	1.1	141	240
Naraha Town, Yamadaoka	37.24358	140.9665	3.2	130		64.0	3.0	217	28.1
Naraha Town, Shimokobana	37.25817	140.9692	5.7	130	47	114	2.5	261	33.9
Tomioka Town, Osuge	37.37439	141.0081	55	5000	1100	28.6	1.3	124	620
Tomioka Town, Motooka	37.34036	140.9807	26	530	93	128	1.5	245	130
Namie Town, Minamitsushima	37.55683	140.7897	4.4	2100		5.5	1.1	90.4	190
Namie Town, Akogi-Teshichiro	37.59606	140.7541	17	7900	920	5.6	1.1	89.9	710
Namie Town, Akogi-Hirusone	37.54186	140.8622	2.8	1300	220	5.6	1.1	91.5	119
Namie Town, Akogi-Kunugidaira	37.56053	140.8238	19	5700	450	8.7	0.8	83.6	477
Namie Town, Akougi-Shiobite	37.56683	140.8021	10	2500		10.4	1.0	91.8	230
Shinchi Town	37.85694	140.8808	0.65	45	9.2	37.6	1.2	130	5.9
Iitate Village, Komiya	37.62881	140.7731	3.2	1300	190	6.4	1.1	92.4	120
Iitate Village, Warabidaira	37.62539	140.8105	3.7	1400		6.9	1.5	103	144.4
Iitate Village, Okura	37.72678	140.8348	0.49	140	29	9.1	1.3	102	14.2
Iitate Village, Notegami	37.63806	140.7982	5.3	1500		9.2	1.3	103	154
Iitate Village, Matsuzuka	37.68981	140.7201	3.6	1000		9.4	1.1	94.5	94.5
Iitate Village, Kusano	37.71753	140.7633	1.2	300		10.4	1.4	106	31.6
Iitate Village, Iitoi	37.66136	140.6972	1.5	280		13.9	1.0	97.6	27.3
Soma City, Nokikitahara	37.84108	140.8961	0.064	52	8.8	3.2	1.1	87.8	4.6
Soma City, Otsubo-Maenosawa	37.82283	140.8953	0.2	55	11	9.5	1.1	95.3	5.2
Soma City, Hatsuno-Nishihara	37.82603	140.8707	0.23	27	3.3	22.2	0.9	104	2.8
Miharu Town, Nanakusagi	37.475	140.4906	0.56	100	22	14.6	0.9	94.1	9.4
Miharu Town, Omachi	37.44297	140.4891	0.48	64	12	19.5	1.1	106	6.8
Ono Town	37.30439	140.6186	0.11	23	3.5	12.4	0.7	87.4	2.0
Tanura City, Miyakoji	37.45814	140.7183	0.24	160	16	3.9	0.8	79.8	12.8
Tanura City, Funehiki	37.51436	140.6562	0.68	88		20.1	1.2	109	9.6
Samekawa Village	37.07261	140.4606	0.14	19	2.7	19.2	0.9	99.6	1.9
Tanakura Town	37.01122	140.3368	0.22	76	2.8	7.5	0.8	82.8	6.3
Minami-Soma City, Takanokura	37.62867	140.8983	0.77	250		8.0	2.4	133	33.3
Minami-Soma City, Haramachi	37.62383	140.9612	0.42	100	25	10.9	1.4	105	10.5
Minami-Soma City, Karasuzaki	37.68547	141.0106	0.15	6.8		57.4	1.4	160	1.1
Nihonmatsu City, Kawasaki	37.61136	140.4875	0.63	270		6.1	1.0	88.3	23.8
Nihonmatsu City, Hatsumori	37.52533	140.5335	0.7	180	32	10.1	1.1	96.2	17.3
Nihonmatsu City, Babadaira	37.57969	140.3518	0.26	56		12.1	1.4	107	6.0
Nihonmatsu City, Dake-Onsen	37.60419	140.3556	0.28	29	8.7	25.1	1.7	131	3.8
Fukushima City, Arai	37.71081	140.3873	0.18	39		12.0	1.2	100	3.9
Fukushima City, Watari	37.72	140.4994	1.7	400		11.1	1.4	106	42.5
Fukushima City, Onami	37.757083	140.55381	2.9	570	110	13.2	1.4	108	61.3
Fukushima City, Matsukawacho	37.68006	140.3562	0.41	67		15.9	1.2	105	7.0
Motomiya City, Motomiya	37.52286	140.4116	0.12	92	6.5	3.4	0.8	78.3	7.2
Motomiya City, Arai	37.48628	140.3833	0.33	24	6.6	35.8	1.3	131	3.2

The ratios decay-corrected to 15 March 2011. The ^129m^Te/^137^Cs ratios interpolated with a multilevel B spline by SAGA-GIS [10] are listed.

**Fig. 5.**
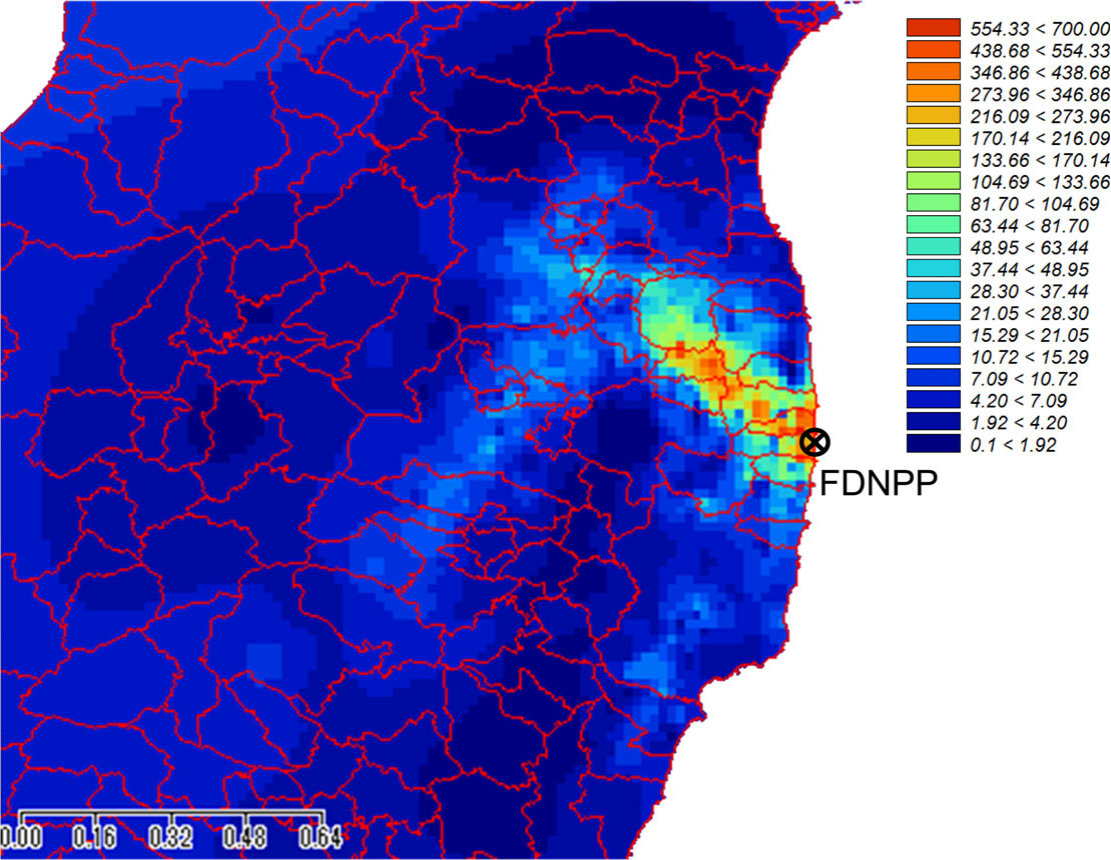
Map of the cumulative β-ray dose (mSv) on ground surface in the first year after deposition.

The map of the estimated cumulative soil surface β-ray dose is shown in Fig. 5, edited by interpolating the results with the multilevel B spline interpolation using SAGA-GIS [10]. Three higher cumulative β-ray dose regions can be clearly seen in the Akogi-Teshichiro and Akogi-Kunugidaira regions in Namie Town, and also from Futaba Town to northern Tomioka Town. Compared with the cumulative γ-ray dose map produced by MEXT [11], the β-ray dose is slightly larger than the γ-ray dose around Iwaki City. This is due to the ^129m,129^Te contributions, which have longer half-lives (33.6 days) than ^131^I (8.021 days) and higher β-ray emission rates of ∼90% compared with the γ-ray emission rates (<10%).

As already stated in the Introduction, our estimation used the 70-μm dose equivalent as the skin dose for humans. These estimates are based on the assumption that people stay outside houses or buildings continuously for a year. Therefore, this skin dose is not strictly accurate for humans; however, the doses are fairly accurate for organisms living in the outside environment, such as small insects, plant leaves, etc.

## CONCLUSION

The cumulative soil surface β-ray dose was calculated using the 2-km mesh soil contamination data by MEXT and our previously published β-ray dose calculation technique. From that, an estimated cumulative soil surface β-ray dose map was produced. As a result of this map, areas estimated to have a higher cumulative β-ray dose on the soil surface for the first year after the FDNPP accident were found to be located in the Akogi-Teshichiro to Akogi-Kunugidaira region in Namie Town and from Futaba Town to northern Tomioka Town. The highest estimated cumulative β-ray dose was 710 mSv for one year at Akogi-Teshichiro, Namie Town.

## FUNDING

This study was supported by Grant-in-Aid for Challenging Exploratory Research No. 26550031 from the Japan Society for the Promotion of Science (JSPS). Funding to pay the Open Access publication charges for this special issue was provided by the Grant-in-Aid from the Japan Society for the Promotion of Science (JSPS) [KAKENHI Grant No. 26253022].
